# 3-(2,4-Dichloro­phen­yl)-5-(4-fluoro­phen­yl)-2-phenyl-7-(trifluoro­meth­yl)pyrazolo­[1,5-*a*]pyrimidine

**DOI:** 10.1107/S1600536812023641

**Published:** 2012-05-31

**Authors:** Ju Liu, Zhi-Qiang Cai, Yang Wang, Yu-Li Sang, Li-Feng Xu

**Affiliations:** a College of Pharmacy, Liaoning University, Shenyang 110036, People’s Republic of China; b Panjin Vocational and Technical College, Panjin 120010, People’s Republic of China; c Tianjin Key Laboratory of Molecular Design and Drug Discovery, State Key Laboratory of Drug Delivery Technology and Pharmacokinetics, Tianjin Institute of Pharmaceutical Research, Tianjin 300193, People’s Republic of China

## Abstract

In the title compound, C_25_H_13_Cl_2_F_4_N_3_, there are four planar systems, *viz.* three benzene rings and a pyrazolo­[1,5-*a*]pyrim­idine system [r.m.s. deviation = 0.002 Å]. The dihedral angle between the dichloro­phenyl ring and the unsubstituted phenyl ring is 69.95 (5)°, while that between the fluoro­phenyl ring and the unsubstituted phenyl ring is 7.97 (10)°. The crystal packing is dominated by van der Waals inter­actions. A Cl⋯Cl inter­action of 3.475 (3) Å also occurs.

## Related literature

For background information and the related structures, see: Liu *et al.* (2012 ▶); Frizzo *et al.* (2008 ▶); Bui *et al.* (2009 ▶). For the synthesis of other pyrozolo[1,5-*a*]pyrimidine derivatives and for their pharmacological applications, see: Fraley *et al.* (2012 ▶); Dalinger *et al.* (2005 ▶); Dennis *et al.* (2004 ▶).
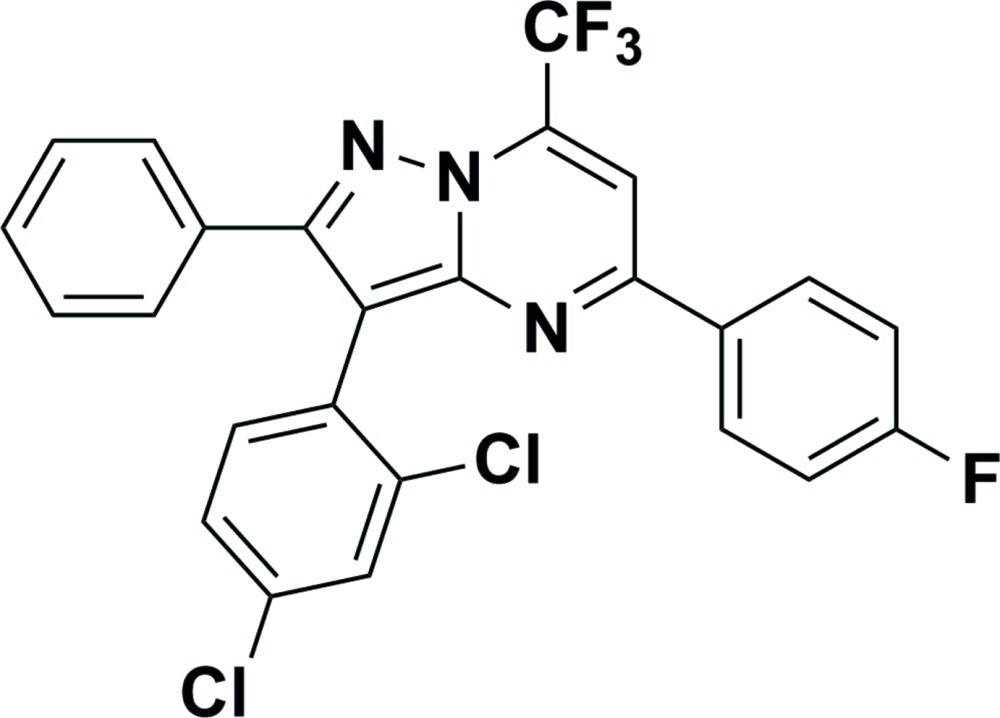


## Experimental

### 

#### Crystal data

C_25_H_13_Cl_2_F_4_N_3_
*M* *_r_* = 502.28
Monoclinic, 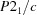

*a* = 9.0826 (18) Å
*b* = 9.0606 (18) Å
*c* = 27.259 (6) Å
β = 99.46 (3)°
*V* = 2212.7 (8) Å^3^
*Z* = 4
Mo *K*α radiation
μ = 0.35 mm^−1^
*T* = 293 K
0.24 × 0.22 × 0.20 mm

#### Data collection

Rigaku Saturn diffractometer
Absorption correction: multi-scan (*CrystalClear*; Rigaku/MSC, 2005 ▶) *T*_min_ = 0.922, *T*_max_ = 0.934
15847 measured reflections
3895 independent reflections
3056 reflections with *I* > 2σ(*I*)
*R*_int_ = 0.041

#### Refinement

*R*[*F* ^2^ > 2σ(*F* ^2^)] = 0.051
*wR*(*F* ^2^) = 0.143
*S* = 1.09
3895 reflections
308 parameters
H-atom parameters constrained
Δρ_max_ = 0.37 e Å^−3^
Δρ_min_ = −0.46 e Å^−3^

### 

Data collection: *RAPID-AUTO* (Rigaku, 1998) ▶; cell refinement: *RAPID-AUTO* ▶; data reduction: *CrystalClear* (Rigaku/MSC, 2005 ▶); program(s) used to solve structure: *SHELXS97* (Sheldrick, 2008 ▶); program(s) used to refine structure: *SHELXL97* (Sheldrick, 2008 ▶); molecular graphics: *SHELXTL* (Sheldrick, 2008 ▶); software used to prepare material for publication: *SHELXTL*.

## Supplementary Material

Crystal structure: contains datablock(s) I, global. DOI: 10.1107/S1600536812023641/kp2419sup1.cif

Structure factors: contains datablock(s) I. DOI: 10.1107/S1600536812023641/kp2419Isup2.hkl

Supplementary material file. DOI: 10.1107/S1600536812023641/kp2419Isup3.cml

Additional supplementary materials:  crystallographic information; 3D view; checkCIF report

## Figures and Tables

**Fig. 1.**
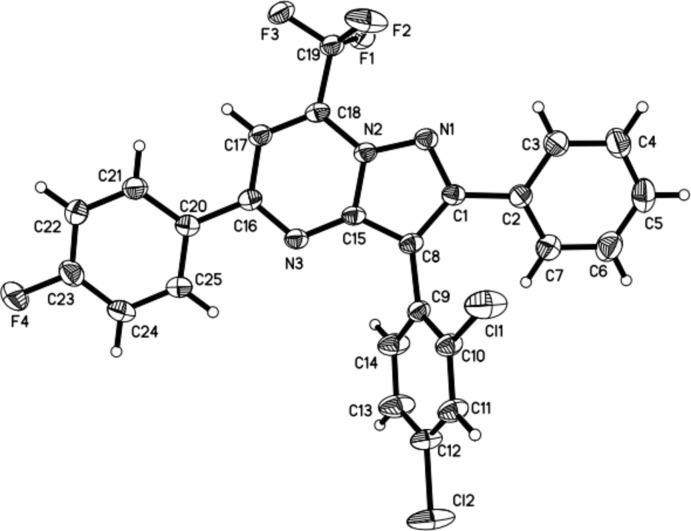
The structure of C25H13Cl2F4N3 with all non-H atom-labelling scheme and ellipsoids drawn at the 50% probability level.

## supplementary crystallographic information

### Comment

The pyrazolo[1,5-*a*]pyrimidine structural motif may be found in a large number of pharmaceutical agents with a diverse range of physiological activities, for example, antiepileptic agents, anxiolytics, antidepressants, agents for treatment of sleep disorders and oncolytics. A series of antagonist of protease-activated PAR2 receptors were reported (Fraley,*et al.*, 2012 and Dalinger,*et al.*, 2005). As a part of our ongoing study of pyrazolo[1,5-*a*]pyrimidine derivatives containing 5-(4-fluorophenyl) and 3-(2,4-dichlorophenyl) substituents (Liu,*et al.*, 2012), we report herein the crystal structure of the title compound.

The title molecule (Fig. 1) bond lengths and angles are generally within normal ranges. The dihedral angles between fluorobenzene ring and benzene ring is 7.97°. The dihedral angle between dichlorophenyl ring and benzene ring is 69.95°. The torsion angles C(16)—C(17)—C(18)—C(19), N(2)—N(1)—C(1)—C(2), C(21)—C(22)—C(23)—F(4) and C(10)—C(11)—C(12)—Cl(2) are -178.71 (19), 175.35 (17), -178.4 (2) and -179.9 (2), respectively. The crystal structure is held together by van der Waals forces and pronounced Cl···Cl interaction of 3.475 (3) Å (Bui, *et al.*, 2009).

### Experimental

A mixture of the corresponding 4-(2,4-dichlorophenyl)-3-phenyl-1*H*-pyrazol-5-amine (1.50 g, 4.93 mmol) and the 4,4,4-trifluoro-1-(4-fluorophenyl)butane-1,3-dione (1.27 g, 5.42 6 mmol) in a flask (25 mL) was heated at 453–458 K for 2.5 h, allowing elimination of the water evolved. After cooling to room temperature, the solid in the flask was recrystallised from methanol to yield the title compound as a yellow solid (1.55 g, 62.58%). Crystals suitable for X-ray analysis were obtained from EtOH : EtOAc(1:1) solution mixture by slow evaporation.

### Refinement

All H atoms were geometrically positioned (C—H 0.93 Å) and treated as riding, with *U*_iso_(H) = 1.2Ueq(C).Due to lack of heavy atoms, Friedel pairs were merged in refinement.

### Crystal data

**Table:**

C_25_H_13_Cl_2_F_4_N_3_	*F*(000) = 1016
*M**_r_* = 502.28	*D*_x_ = 1.508 Mg m^−^^3^
Monoclinic, *P*2_1_/*c*	Mo *K*α radiation, λ = 0.71073 Å
Hall symbol: -P 2ybc	Cell parameters from 5009 reflections
*a* = 9.0826 (18) Å	θ = 2.3–27.9°
*b* = 9.0606 (18) Å	µ = 0.35 mm^−^^1^
*c* = 27.259 (6) Å	*T* = 293 K
β = 99.46 (3)°	Prism, yellow
*V* = 2212.7 (8) Å^3^	0.24 × 0.22 × 0.20 mm
*Z* = 4	

### Data collection

**Table:**

Rigaku Saturn diffractometer	3895 independent reflections
Radiation source: rotating anode	3056 reflections with *I* > 2σ(*I*)
Confocal monochromator	*R*_int_ = 0.041
ω scans	θ_max_ = 25.0°, θ_min_ = 2.4°
Absorption correction: multi-scan (*CrystalClear*; Rigaku/MSC, 2005)	*h* = −10→10
*T*_min_ = 0.922, *T*_max_ = 0.934	*k* = −10→7
15847 measured reflections	*l* = −30→32

### Refinement

**Table:**

Refinement on *F*^2^	Secondary atom site location: difference Fourier map
Least-squares matrix: full	Hydrogen site location: inferred from neighbouring sites
*R*[*F*^2^ > 2σ(*F*^2^)] = 0.051	H-atom parameters constrained
*wR*(*F*^2^) = 0.143	*w* = 1/[σ^2^(*F*_o_^2^) + (0.0833*P*)^2^ + 0.040*P*] where *P* = (*F*_o_^2^ + 2*F*_c_^2^)/3
*S* = 1.09	(Δ/σ)_max_ = 0.001
3895 reflections	Δρ_max_ = 0.37 e Å^−^^3^
308 parameters	Δρ_min_ = −0.46 e Å^−^^3^
0 restraints	Extinction correction: *SHELXL97* (Sheldrick, 2008), Fc^*^=kFc[1+0.001xFc^2^λ^3^/sin(2θ)]^-1/4^
Primary atom site location: structure-invariant direct methods	Extinction coefficient: 0.028 (3)

### Special details

**Table:**

Geometry. All e.s.d.'s (except the e.s.d. in the dihedral angle between two l.s. planes) are estimated using the full covariance matrix. The cell e.s.d.'s are taken into account individually in the estimation of e.s.d.'s in distances, angles and torsion angles; correlations between e.s.d.'s in cell parameters are only used when they are defined by crystal symmetry. An approximate (isotropic) treatment of cell e.s.d.'s is used for estimating e.s.d.'s involving l.s. planes.
Refinement. Refinement of *F*^2^ against ALL reflections. The weighted *R*-factor *wR* and goodness of fit *S* are based on *F*^2^, conventional *R*-factors *R* are based on *F*, with *F* set to zero for negative *F*^2^. The threshold expression of *F*^2^ > σ(*F*^2^) is used only for calculating *R*-factors(gt) *etc*. and is not relevant to the choice of reflections for refinement. *R*-factors based on *F*^2^ are statistically about twice as large as those based on *F*, and *R*- factors based on ALL data will be even larger.

### Fractional atomic coordinates and isotropic or equivalent isotropic displacement parameters (Å^2^)

**Table:**

	*x*	*y*	*z*	*U*_iso_*/*U*_eq_	
Cl1	1.31929 (9)	0.44828 (10)	0.47969 (3)	0.0868 (3)	
Cl2	0.94948 (12)	0.20741 (11)	0.59085 (3)	0.1024 (4)	
F1	1.09069 (17)	0.35785 (15)	0.20644 (5)	0.0619 (4)	
F2	1.2102 (2)	0.5478 (2)	0.23496 (6)	0.0886 (6)	
F3	0.9955 (2)	0.56762 (17)	0.18909 (5)	0.0831 (6)	
F4	0.24299 (18)	0.7892 (2)	0.32444 (6)	0.0917 (6)	
N1	1.2012 (2)	0.33960 (19)	0.31669 (6)	0.0413 (4)	
N2	1.0698 (2)	0.41358 (18)	0.31156 (6)	0.0385 (4)	
N3	0.8699 (2)	0.47095 (19)	0.35650 (6)	0.0401 (4)	
C1	1.2191 (2)	0.2873 (2)	0.36384 (7)	0.0388 (5)	
C2	1.3488 (2)	0.1903 (2)	0.37979 (8)	0.0432 (5)	
C3	1.4710 (3)	0.1954 (3)	0.35534 (9)	0.0542 (6)	
H3	1.4737	0.2649	0.3304	0.065*	
C4	1.5895 (3)	0.0981 (3)	0.36768 (10)	0.0670 (8)	
H4	1.6707	0.1023	0.3509	0.080*	
C5	1.5865 (3)	−0.0044 (3)	0.40471 (11)	0.0727 (9)	
H5	1.6655	−0.0701	0.4129	0.087*	
C6	1.4674 (4)	−0.0098 (3)	0.42954 (11)	0.0731 (8)	
H6	1.4664	−0.0782	0.4549	0.088*	
C7	1.3487 (3)	0.0857 (3)	0.41720 (9)	0.0594 (7)	
H7	1.2678	0.0802	0.4341	0.071*	
C8	1.1017 (2)	0.3286 (2)	0.38894 (7)	0.0395 (5)	
C9	1.0711 (2)	0.2977 (2)	0.43952 (7)	0.0402 (5)	
C10	1.1586 (3)	0.3478 (2)	0.48288 (8)	0.0468 (6)	
C11	1.1222 (3)	0.3204 (3)	0.52918 (8)	0.0553 (7)	
H11	1.1831	0.3551	0.5576	0.066*	
C12	0.9960 (3)	0.2419 (3)	0.53295 (8)	0.0592 (7)	
C13	0.9050 (3)	0.1923 (3)	0.49108 (10)	0.0747 (9)	
H13	0.8181	0.1407	0.4936	0.090*	
C14	0.9437 (3)	0.2199 (3)	0.44528 (9)	0.0620 (7)	
H14	0.8820	0.1849	0.4170	0.074*	
C15	1.0032 (2)	0.4081 (2)	0.35423 (7)	0.0378 (5)	
C16	0.8006 (2)	0.5390 (2)	0.31605 (7)	0.0386 (5)	
C17	0.8643 (3)	0.5463 (2)	0.27171 (8)	0.0424 (5)	
H17	0.8129	0.5937	0.2438	0.051*	
C18	0.9989 (2)	0.4847 (2)	0.27000 (7)	0.0394 (5)	
C19	1.0751 (3)	0.4900 (3)	0.22528 (8)	0.0461 (6)	
C20	0.6539 (2)	0.6071 (2)	0.31877 (7)	0.0396 (5)	
C21	0.5912 (3)	0.7106 (3)	0.28349 (9)	0.0493 (6)	
H21	0.6427	0.7381	0.2581	0.059*	
C22	0.4542 (3)	0.7726 (3)	0.28576 (9)	0.0573 (7)	
H22	0.4130	0.8419	0.2623	0.069*	
C23	0.3796 (3)	0.7307 (3)	0.32307 (10)	0.0576 (7)	
C24	0.4372 (3)	0.6316 (3)	0.35902 (9)	0.0564 (6)	
H24	0.3848	0.6061	0.3844	0.068*	
C25	0.5753 (3)	0.5702 (2)	0.35670 (8)	0.0473 (6)	
H25	0.6163	0.5030	0.3809	0.057*	

### Atomic displacement parameters (Å^2^)

**Table:**

	*U*^11^	*U*^22^	*U*^33^	*U*^12^	*U*^13^	*U*^23^
Cl1	0.0765 (6)	0.1313 (7)	0.0511 (4)	−0.0570 (5)	0.0062 (4)	−0.0089 (4)
Cl2	0.1446 (9)	0.1281 (8)	0.0411 (4)	−0.0592 (6)	0.0350 (5)	−0.0028 (4)
F1	0.0830 (11)	0.0617 (9)	0.0432 (8)	0.0200 (7)	0.0171 (7)	−0.0052 (6)
F2	0.0899 (13)	0.1194 (15)	0.0648 (10)	−0.0465 (11)	0.0371 (9)	−0.0103 (9)
F3	0.1145 (14)	0.0927 (12)	0.0507 (9)	0.0534 (10)	0.0388 (9)	0.0330 (8)
F4	0.0567 (10)	0.1403 (16)	0.0836 (12)	0.0343 (10)	0.0276 (9)	0.0172 (11)
N1	0.0423 (11)	0.0467 (10)	0.0346 (9)	−0.0032 (8)	0.0056 (8)	−0.0020 (8)
N2	0.0449 (11)	0.0409 (10)	0.0303 (9)	0.0002 (8)	0.0075 (8)	0.0001 (7)
N3	0.0425 (10)	0.0471 (10)	0.0315 (9)	−0.0027 (8)	0.0083 (8)	−0.0011 (8)
C1	0.0382 (12)	0.0458 (12)	0.0321 (11)	−0.0077 (9)	0.0049 (9)	−0.0040 (9)
C2	0.0425 (13)	0.0500 (13)	0.0356 (11)	−0.0023 (10)	0.0021 (10)	−0.0076 (10)
C3	0.0491 (14)	0.0662 (15)	0.0459 (14)	−0.0023 (12)	0.0033 (11)	−0.0048 (12)
C4	0.0484 (16)	0.087 (2)	0.0648 (17)	0.0074 (14)	0.0059 (13)	−0.0123 (16)
C5	0.0590 (19)	0.0739 (18)	0.080 (2)	0.0197 (15)	−0.0047 (16)	−0.0118 (16)
C6	0.080 (2)	0.0664 (17)	0.0702 (19)	0.0159 (16)	0.0042 (16)	0.0117 (15)
C7	0.0614 (16)	0.0612 (15)	0.0560 (16)	0.0081 (13)	0.0108 (13)	0.0055 (12)
C8	0.0430 (12)	0.0447 (12)	0.0299 (10)	−0.0063 (9)	0.0036 (9)	−0.0008 (9)
C9	0.0408 (12)	0.0465 (12)	0.0320 (11)	−0.0018 (10)	0.0028 (9)	0.0022 (9)
C10	0.0486 (14)	0.0560 (14)	0.0344 (12)	−0.0109 (11)	0.0024 (10)	−0.0003 (10)
C11	0.0632 (16)	0.0666 (16)	0.0337 (12)	−0.0140 (13)	0.0010 (11)	−0.0032 (11)
C12	0.0747 (18)	0.0703 (16)	0.0346 (13)	−0.0133 (14)	0.0148 (12)	0.0029 (11)
C13	0.0732 (19)	0.104 (2)	0.0478 (15)	−0.0433 (17)	0.0131 (14)	0.0027 (15)
C14	0.0591 (16)	0.0897 (19)	0.0355 (13)	−0.0285 (14)	0.0028 (11)	−0.0048 (12)
C15	0.0424 (12)	0.0442 (12)	0.0274 (10)	−0.0045 (9)	0.0071 (9)	−0.0028 (9)
C16	0.0439 (12)	0.0378 (11)	0.0338 (11)	−0.0055 (9)	0.0052 (9)	−0.0028 (9)
C17	0.0494 (14)	0.0440 (12)	0.0341 (11)	0.0012 (10)	0.0074 (10)	0.0014 (9)
C18	0.0518 (14)	0.0371 (11)	0.0303 (11)	−0.0035 (10)	0.0095 (10)	−0.0006 (9)
C19	0.0576 (15)	0.0468 (13)	0.0358 (12)	0.0053 (11)	0.0133 (11)	0.0039 (10)
C20	0.0407 (12)	0.0421 (12)	0.0362 (11)	−0.0068 (9)	0.0064 (9)	−0.0043 (9)
C21	0.0441 (13)	0.0639 (15)	0.0404 (12)	−0.0012 (11)	0.0080 (10)	0.0047 (11)
C22	0.0495 (14)	0.0713 (16)	0.0504 (14)	0.0128 (12)	0.0064 (12)	0.0111 (12)
C23	0.0412 (14)	0.0759 (17)	0.0563 (15)	0.0061 (12)	0.0093 (12)	−0.0060 (13)
C24	0.0552 (15)	0.0684 (16)	0.0504 (14)	−0.0040 (13)	0.0227 (12)	−0.0013 (13)
C25	0.0540 (14)	0.0510 (13)	0.0389 (12)	−0.0036 (11)	0.0134 (11)	0.0012 (10)

### Geometric parameters (Å, º)

**Table:**

Cl1—C10	1.734 (2)	C8—C9	1.477 (3)
Cl2—C12	1.728 (2)	C9—C14	1.386 (3)
F1—C19	1.320 (3)	C9—C10	1.387 (3)
F2—C19	1.320 (3)	C10—C11	1.379 (3)
F3—C19	1.325 (2)	C11—C12	1.367 (3)
F4—C23	1.356 (3)	C11—H11	0.9300
N1—C1	1.354 (3)	C12—C13	1.370 (3)
N1—N2	1.356 (2)	C13—C14	1.375 (3)
N2—C18	1.369 (3)	C13—H13	0.9300
N2—C15	1.397 (3)	C14—H14	0.9300
N3—C16	1.328 (3)	C16—C17	1.425 (3)
N3—C15	1.349 (3)	C16—C20	1.481 (3)
C1—C8	1.409 (3)	C17—C18	1.352 (3)
C1—C2	1.477 (3)	C17—H17	0.9300
C2—C3	1.386 (3)	C18—C19	1.498 (3)
C2—C7	1.392 (3)	C20—C25	1.390 (3)
C3—C4	1.389 (4)	C20—C21	1.396 (3)
C3—H3	0.9300	C21—C22	1.376 (3)
C4—C5	1.375 (4)	C21—H21	0.9300
C4—H4	0.9300	C22—C23	1.365 (4)
C5—C6	1.369 (4)	C22—H22	0.9300
C5—H5	0.9300	C23—C24	1.368 (4)
C6—C7	1.380 (4)	C24—C25	1.384 (3)
C6—H6	0.9300	C24—H24	0.9300
C7—H7	0.9300	C25—H25	0.9300
C8—C15	1.391 (3)		
			
C1—N1—N2	103.71 (17)	C12—C13—H13	120.4
N1—N2—C18	127.17 (18)	C14—C13—H13	120.4
N1—N2—C15	112.99 (16)	C13—C14—C9	122.6 (2)
C18—N2—C15	119.81 (18)	C13—C14—H14	118.7
C16—N3—C15	117.66 (18)	C9—C14—H14	118.7
N1—C1—C8	112.68 (18)	N3—C15—C8	131.98 (19)
N1—C1—C2	116.95 (19)	N3—C15—N2	122.52 (18)
C8—C1—C2	130.22 (19)	C8—C15—N2	105.50 (19)
C3—C2—C7	118.2 (2)	N3—C16—C17	121.5 (2)
C3—C2—C1	120.1 (2)	N3—C16—C20	117.33 (19)
C7—C2—C1	121.6 (2)	C17—C16—C20	121.14 (19)
C2—C3—C4	120.8 (2)	C18—C17—C16	120.4 (2)
C2—C3—H3	119.6	C18—C17—H17	119.8
C4—C3—H3	119.6	C16—C17—H17	119.8
C5—C4—C3	119.9 (3)	C17—C18—N2	118.08 (19)
C5—C4—H4	120.0	C17—C18—C19	123.80 (19)
C3—C4—H4	120.0	N2—C18—C19	118.1 (2)
C6—C5—C4	119.9 (3)	F1—C19—F2	106.4 (2)
C6—C5—H5	120.0	F1—C19—F3	105.79 (17)
C4—C5—H5	120.0	F2—C19—F3	108.3 (2)
C5—C6—C7	120.4 (3)	F1—C19—C18	112.40 (18)
C5—C6—H6	119.8	F2—C19—C18	112.87 (18)
C7—C6—H6	119.8	F3—C19—C18	110.76 (19)
C6—C7—C2	120.7 (3)	C25—C20—C21	118.2 (2)
C6—C7—H7	119.6	C25—C20—C16	120.87 (19)
C2—C7—H7	119.6	C21—C20—C16	120.9 (2)
C15—C8—C1	105.08 (18)	C22—C21—C20	120.9 (2)
C15—C8—C9	122.3 (2)	C22—C21—H21	119.6
C1—C8—C9	132.56 (19)	C20—C21—H21	119.6
C14—C9—C10	116.2 (2)	C23—C22—C21	118.9 (2)
C14—C9—C8	119.36 (18)	C23—C22—H22	120.6
C10—C9—C8	124.3 (2)	C21—C22—H22	120.6
C11—C10—C9	122.1 (2)	F4—C23—C22	118.7 (2)
C11—C10—Cl1	118.08 (17)	F4—C23—C24	118.7 (2)
C9—C10—Cl1	119.85 (17)	C22—C23—C24	122.6 (2)
C12—C11—C10	119.5 (2)	C23—C24—C25	118.3 (2)
C12—C11—H11	120.2	C23—C24—H24	120.9
C10—C11—H11	120.2	C25—C24—H24	120.9
C11—C12—C13	120.4 (2)	C24—C25—C20	121.2 (2)
C11—C12—Cl2	119.78 (19)	C24—C25—H25	119.4
C13—C12—Cl2	119.8 (2)	C20—C25—H25	119.4
C12—C13—C14	119.2 (2)		
			
C1—N1—N2—C18	−178.37 (19)	C1—C8—C15—N3	177.8 (2)
C1—N1—N2—C15	−0.6 (2)	C9—C8—C15—N3	−0.1 (3)
N2—N1—C1—C8	−0.7 (2)	C1—C8—C15—N2	−2.0 (2)
N2—N1—C1—C2	175.35 (17)	C9—C8—C15—N2	−179.81 (18)
N1—C1—C2—C3	22.1 (3)	N1—N2—C15—N3	−178.07 (17)
C8—C1—C2—C3	−162.6 (2)	C18—N2—C15—N3	−0.1 (3)
N1—C1—C2—C7	−153.9 (2)	N1—N2—C15—C8	1.7 (2)
C8—C1—C2—C7	21.4 (3)	C18—N2—C15—C8	179.63 (17)
C7—C2—C3—C4	0.4 (3)	C15—N3—C16—C17	−0.2 (3)
C1—C2—C3—C4	−175.6 (2)	C15—N3—C16—C20	179.71 (17)
C2—C3—C4—C5	−0.3 (4)	N3—C16—C17—C18	−0.7 (3)
C3—C4—C5—C6	−0.4 (4)	C20—C16—C17—C18	179.40 (18)
C4—C5—C6—C7	1.0 (4)	C16—C17—C18—N2	1.2 (3)
C5—C6—C7—C2	−0.8 (4)	C16—C17—C18—C19	−178.71 (19)
C3—C2—C7—C6	0.1 (4)	N1—N2—C18—C17	176.86 (18)
C1—C2—C7—C6	176.1 (2)	C15—N2—C18—C17	−0.8 (3)
N1—C1—C8—C15	1.8 (2)	N1—N2—C18—C19	−3.3 (3)
C2—C1—C8—C15	−173.7 (2)	C15—N2—C18—C19	179.10 (19)
N1—C1—C8—C9	179.3 (2)	C17—C18—C19—F1	−115.6 (2)
C2—C1—C8—C9	3.9 (4)	N2—C18—C19—F1	64.6 (3)
C15—C8—C9—C14	59.2 (3)	C17—C18—C19—F2	124.1 (2)
C1—C8—C9—C14	−118.0 (3)	N2—C18—C19—F2	−55.8 (3)
C15—C8—C9—C10	−117.6 (2)	C17—C18—C19—F3	2.5 (3)
C1—C8—C9—C10	65.3 (3)	N2—C18—C19—F3	−177.34 (19)
C14—C9—C10—C11	0.7 (4)	N3—C16—C20—C25	−15.9 (3)
C8—C9—C10—C11	177.6 (2)	C17—C16—C20—C25	164.1 (2)
C14—C9—C10—Cl1	−179.21 (19)	N3—C16—C20—C21	163.79 (19)
C8—C9—C10—Cl1	−2.3 (3)	C17—C16—C20—C21	−16.3 (3)
C9—C10—C11—C12	−0.2 (4)	C25—C20—C21—C22	−1.1 (3)
Cl1—C10—C11—C12	179.8 (2)	C16—C20—C21—C22	179.3 (2)
C10—C11—C12—C13	−0.9 (4)	C20—C21—C22—C23	−0.3 (4)
C10—C11—C12—Cl2	−179.9 (2)	C21—C22—C23—F4	−178.4 (2)
C11—C12—C13—C14	1.3 (5)	C21—C22—C23—C24	1.4 (4)
Cl2—C12—C13—C14	−179.7 (2)	F4—C23—C24—C25	178.7 (2)
C12—C13—C14—C9	−0.7 (5)	C22—C23—C24—C25	−1.1 (4)
C10—C9—C14—C13	−0.3 (4)	C23—C24—C25—C20	−0.3 (4)
C8—C9—C14—C13	−177.3 (3)	C21—C20—C25—C24	1.4 (3)
C16—N3—C15—C8	−179.1 (2)	C16—C20—C25—C24	−179.0 (2)
C16—N3—C15—N2	0.6 (3)		
